# Prevalence of premenstrual syndrome and its associated factors in Africa: a systematic review and meta-analysis

**DOI:** 10.3389/fpsyt.2024.1338304

**Published:** 2024-01-31

**Authors:** Fantahun Andualem, Mamaru Melkam, Girmaw Medfu Takelle, Girum Nakie, Techilo Tinsae, Setegn Fentahun, Gidey Rtbey, Jemal Seid, Getachew Muluye Gedef, Desalegn Anmut Bitew, Tilahun Nega Godana

**Affiliations:** ^1^ Department of Psychiatry, College of Medicine and Health Science, University of Gondar, Gondar, Ethiopia; ^2^ Department of Psychiatry College of Medicine and Health Science, Wollo University, Dessie, Ethiopia; ^3^ Department of General Midwifery, College of Medicine and Health Science, University of Gondar, Gondar, Ethiopia; ^4^ Department of Reproductive Health, Institute of Public Health, College of Medicine and Health Science, University of Gondar, Gondar, Ethiopia; ^5^ Department of Internal Medicine, University of Gondar College of Medicine and Health Science, Comprehensive Specialized Hospital, Gondar, Ethiopia

**Keywords:** Africa, prevalence, meta-analysis, premenstrual syndrome, PMS, systematic review

## Abstract

**Background:**

Clinical research and epidemiological studies have shown that many women experience physical and behavioral symptoms that begin during the luteal phase of the menstrual cycle and terminate around the onset of menses; this is called premenstrual syndrome. The reviews stated that the pooled prevalence of premenstrual syndrome was around 50 percent. However, there has been no review done on premenstrual syndrome in Africa. Therefore, the aim of this systematic review and meta-analysis was to summarize the most recent data evidence on the pooled prevalence of premenstrual syndrome and its pooled effect of associated factors in Africa.

**Method:**

We used an appropriate guideline for systematic reviews and meta-analyses reports, which is the Preferred Reporting Items for Systematic Reviews and Meta-Analyses (PRISMA). This review protocol was registered in PROSPERO (CRD42023414021). The publications were identified from PubMed/Medline, EMBASE, Scopus databases, and other grey searches. The included papers were the original data that reported the prevalence of premenstrual syndrome and associated factors published, in English, and papers available online from January 1, 2000, to May 30, 2023. The data was extracted in Microsoft Excel, and then it would be imported into STATA 11.0 for analysis.

**Results:**

We have included 16 studies conducted in African countries with 6530 study participants. In this meta-analysis, the pooled prevalence of premenstrual syndrome among the reproductive-age participants in Africa was 46.98 (95% CI: 28.9–65.06%). Further, in subgroup analysis, the pooled prevalence of premenstrual syndrome was 57.32% in Nigeria, 43.8% in Ethiopia, and 38.6% among university students and 66.04% among secondary school students. Among associated factors, the early age of menarche was significantly related to premenstrual syndrome.

**Conclusion:**

In this review, the pooled prevalence of premenstrual syndrome in Africa was high. Among factors, the early age of menarche was a risk factor for premenstrual syndrome. This finding might help the stakeholders (mental health policy makers, administrators, and mental health professionals) to address prevention, early screening, and management of PMS among reproductive-age women, and to give attention to more vulnerable bodies.

**Systematic review registration:**

https://www.crd.york.ac.uk/PROSPERO, identifier CRD42023414021.

## Introduction

Clinical research and epidemiological studies have shown that many women experience recurrent physical, behavioral, and/or psychological signs and symptoms that begin during the luteal phase of the menstrual cycle and terminate around the onset of menses or shortly thereafter; this is called premenstrual syndrome and/or premenstrual dysphoric disorder (1–3). These signs and symptoms are characterized as; Behavioural and/or psychological signs and symptoms: affective lability (feeling suddenly sad or tearful, or increased sensitivity to rejection), irritability or anger, depressed mood, feelings of hopelessness, anxiety, tension, restlessness, decreased interest in usual activities, lethargy, difficulty in concentration, changes in appetite, and hypersomnia or insomnia. Physical signs and symptoms include breast tenderness or swelling, joint or muscle pain, a sensation of “bloating” or weight gain, headaches or migraines, and abdominal cramps (2, 3).

The severe form of premenstrual syndrome is called premenstrual dysphoric disorder (PMDD) (4). Premenstrual dysphoric disorder was under depressive disorder not otherwise specified in the Diagnostic and Statistical Manual of Mental Disorder (DSM-IV) (5), but after careful scientific review of the evidence, PMDD is a new diagnosis in the Diagnostic and Statistical Manual of Mental Disorder-5 (DSM-5) (6). Almost 20 years of additional research on this condition has confirmed a specific and treatment-responsive form of depressive disorder that begins sometime following ovulation, remits within a few days of menses, and has a marked impact on functioning (6).

The scholars were also strengthened that PMS is a number of signs and symptoms that are characterized by physical symptoms and emotional and behavioral disturbances in the luteal phase of the menstrual cycle in the reproductive age women (7–10). Reproductive age in women (from menarche to menopause) has a different range, but mostly it falls between 15 and 49 years (11, 12). The study showed that an estimated 90% of women of reproductive age suffered from mild to acute premenstrual symptoms. Among them, about 20 to 40 percent encounter moderate to severe symptoms (PMS), while 2 to 8 percent experience severe PMS/PMDD (13). Another study revealed that 95% of women experience PMS; of them, 5% suffered severe PMS (14). In the United States of America (USA), the 12-month prevalence of PMDD is between 1.8% and 5.8% of menstruating women (15). And also, the systematic review and meta-analysis studies revealed that the prevalence of PMS was 47.8% (7) in the global population, PMS (43%), and PMDD (8%) (16) in India, whereas in Ethiopia, more than half (53%) (17) and (54.5%) (18) of the reproductive-age women had PMS and PMDD, respectively. However, there has been no review done on PMS and/or PMDD in Africa. Indeed, PMS and/or PMDD status has been the topic of a large number of studies, with a large variation in reported prevalence rates from 6.1% (19) to 94.8% (20).

The precise cause of PMS is unknown, but research has indicated that changing hormone levels such as those of oestrogen, progesterone, testosterone, prolactin, and serotonin synthesis in the brain also appear to play a major part in PMS (3, 15) (21). Stress, a history of interpersonal trauma, seasonal variations, and sociocultural features of female sexual behavior in general and female gender role in particular are environmental factors linked to the expression of premenstrual dysphoric disorder (6). Premenstrual dysphoric disorder’s heritability in the United States is uncertain. Premenstrual symptoms, on the other hand, have heritability estimates ranging from 30% to 80%, with the most stable component considered to be roughly 50% heritable (6). And also, the review stated that alcohol intake was significantly associated with PMS (22). Whereas, other reviews showed that PMS/PMDD was associated with a higher risk of post-partum depression (PPD) (23), and suicidality (24, 25). Women with PMS or PMDD suffered distress and impairment in interpersonal or workplace functioning. These decreased productivity levels at work and school affect quality of life, increase health care utilization, and can lead to at least 2 days per month of absenteeism at work and an increase in medical appointments (26–28).

Identifying risk factors and the pooled prevalence of MPS could help health care professionals and policymakers plan health promotion, prevention, and early intervention programs. However, per our search, there has been no systematic review or meta-analysis of the epidemiology of PMS in Africa. So, in response to this research gap, the aim of this study is to summarize the most recent data evidence from January 2000 to May 2023 for reproductive-age women in Africa. What is the pooled prevalence of PMS and the pooled effects of associated factors?

## Materials and methods

### Study design

In this study, we followed an appropriate guideline for systematic reviews and meta-analyses reports: of the Preferred Reporting Items for Systematic Reviews and Meta-Analyses (PRISMA) (29) (**Supplementary File 1**). This review protocol was registered in PROSPERO (CRD42023414021).

### Searching strategy

The publications were searched by PubMed/Medline, EMBASE, Cochrane Library, Scopus, HINARI, PsycINFO, African Journals Online (AJOL), and also the grey literature was searched by Google Scholar and World Health Organization (WHO) reports. We used the following search terms (“prevalence” OR “epidemiology” OR “magnitude” AND “premenstrual syndrome” OR “premenstrual dysphoric disorder” AND “associated factors” OR “risk factors” AND “women” AND “Africa”) (**Supplementary File 2**).

### Eligibility criteria

**Figure 1:**
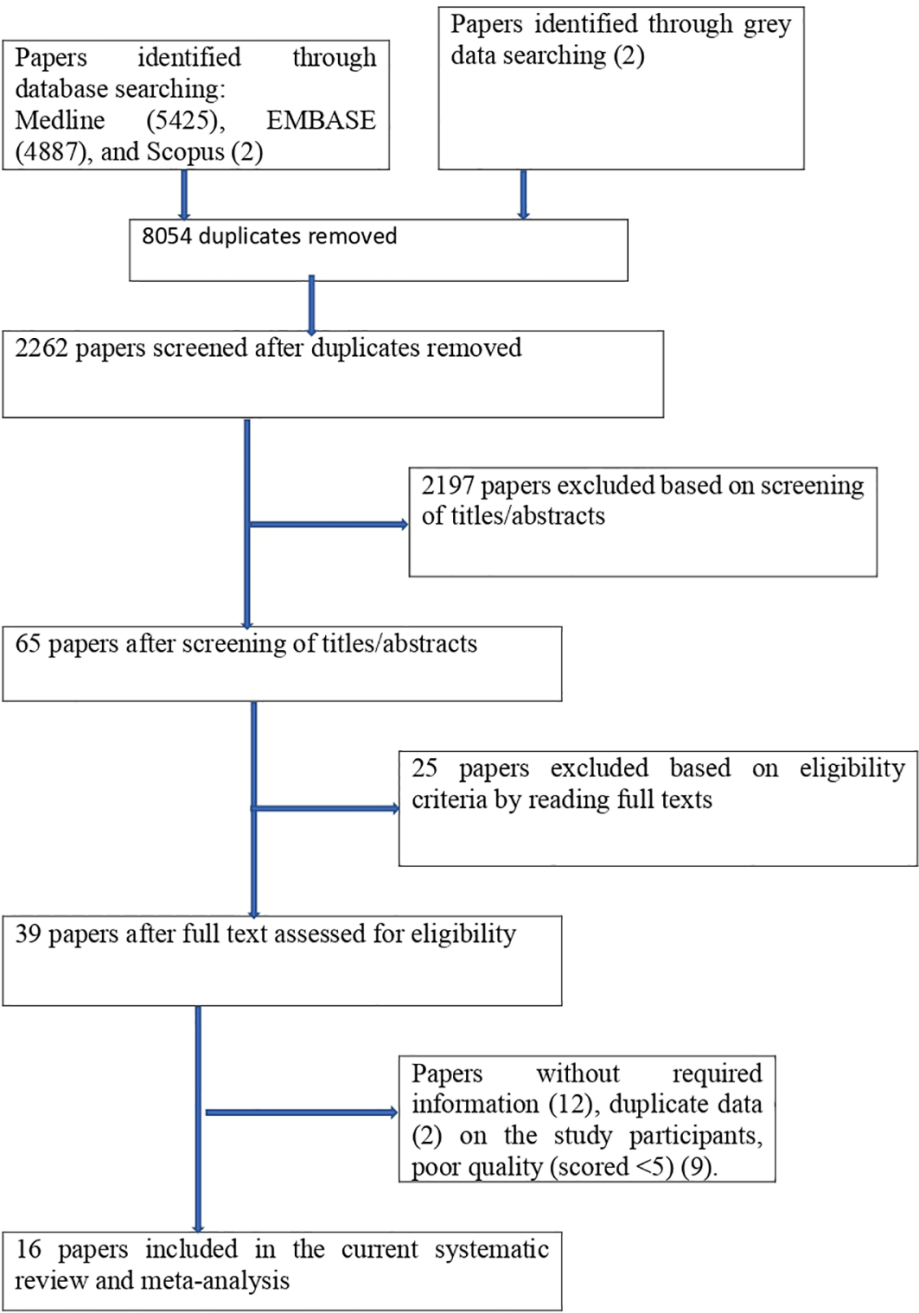
PRISMA flowchart of review search on the prevelance and associated factors of PMS.

The studies had the following criteria: they were done in African countries and were all relevant observational studies (cross-sectional). And also, published in English, published and unpublished articles were considered. Searching was performed from 1 February 2023 to 30 May 2023, and articles available online from 1 January 2000 to 30 May 2023 were considered, but studies that could not be fully accessed (conference abstracts) were excluded. We looked over each study’s abstract and title before adding those to our meta-analysis. Following the selection of pertinent research, the entire text was examined. As seen in **Figure 1**, we did not include any article with no interesting variables in our study.

### Data extraction

MM and GN independently extracted all the necessary data from the articles using a standardized data extraction format. The data extraction format included the following items: the first author’s name, publication year, country where the study was conducted, a screening tool used to examine PMS, number of participants, prevalence of PMS, and associated factors with PMS. The data extraction format was in the form of a two-by-two table. A cross-check was done by MM and GM authors. If contrasting results occurred between the two authors during data extraction, they were discussing ways to achieve consensus and double extraction with other authors.

### Tools and validity

Among the sixteen studies reporting estimates of the prevalence of PMS, six were assessed using the Diagnostic and Statistical Manual of Mental Disorders, fourth edition (DSM-IV). Three studies were assessed using the Premenstrual Syndrome Scale (PMSS), with an inter-rater reliability between 0.81 and 0.97 (30). The PMSS consists of 40 items on a 5-point Likert-type scale (as “never” was scored as “1”, rarely as “2”, sometimes as “3”, very often as “4”, and always as “5” points). The scale score is 40 to 200. A total score estimated at 80 points or above indicates PMS, and an increase in the scores indicates an increase in PMS severity (30, 31).

The American College of Obstetricians and Gynecologist diagnostic criteria (ACOG) were used to assess PMS in two studies (32), where the following criteria were followed:

1. The patient reports one or more of the following affective and somatic symptoms five days before menses in each of the three prior menstrual cycles: affective, depression, angry outbursts, anxiety, irritability, confusion, social withdrawal, somatic, Breast tenderness, abdominal bloating, headache, swelling of the extremities.
2. Symptoms are relieved within 4 days of menses onset without recurrence until at least cycle day 13.
3. Symptoms present in the absence of any pharmacologic therapy, hormone ingestion, or drug or alcohol abuse.
4. Symptoms occur reproducibly during two cycles of prospective recording.
5. The patient suffers from an identifiable dysfunction in social or economic performance.

Another two studies were assessed using the International Classification of Diseases, Tenth Edition (ICD-10). The remaining three studies were measured using a different tool, as shown here: Diagnostic and Statistical Manual of Mental Disorders, Fifth Edition (DSM-5) (6). The premenstrual symptoms screening tool (PSST) was used to assess PMDD, which includes a list of premenstrual psychiatric and physical symptoms and a measure of functional impairment in accordance with DSM-5 criteria (33). And the Calendar of Premenstrual Experiences (COPE) is a valid and reliable tool for the diagnosis of PMS (31, 34, 35). The COPE scoring for each symptom is a 3-point rating scale: absent = 0, present = 1, severe = 2 (31).

### Quality appraisal

The quality of the research reports included in this review was assessed using the Joanna Briggs Institute (JBI) for cross-sectional study quality assessment (36). For this quality assessment tool, it included the following components: the methodological quality of the study, the comparability of the included studies, and the quality of the original articles with respect to statistical analysis. All authors independently evaluated the quality of the original research using JBI. The tool has a total of 9 scores, and articles with medium and high quality (articles that score 5 and above out of a 9-point scale) were included in this review for analysis. Also, some papers were excluded due to low quality scores (that scored less than 5). If there were any discrepancies between authors during the quality assessment of the included studies, they were solved by the involvement of other authors.

### Data processing and analysis

The data was extracted in Microsoft Excel, and then it will be imported into STATA 11.0 for analysis. The extracted data were shown using texts, tables, and forest plots. The standard error of prevalence for each primary or original study was analyzed using the binomial distribution. The prevalence of the original studies was checked for heterogeneity using a heterogeneity I-squire (I^2^) test (37). We used a random-effects meta-analysis model to estimate Der Simonian and Laird’s pooled effect of PMS. And we made a leave-one-out sensitivity analysis to identify the possible source of heterogeneity in the pooled meta-analysis of the prevalence of PMS among women in African countries. Publication bias was detected using funnel plot analysis (38) and Egger - weighted regression tests (39). A p value of less than 5% significance in the Egger test was considered to have statistically significant publication bias (39), Subgroup analysis was done to identify the impact of factors in a particular group for the prediction of the pooled prevalence of PMS.

## Results

### Study identification

There were 10314 publications identified in database searches, and another 2 records were added through grey searches. Among them, 8054 were removed due to duplicates; 2197 papers were excluded based on screening of titles/abstracts; 25 papers were excluded based on eligibility criteria by reading full texts; papers were excluded due to (without required information (12), duplicate data (2), and poor quality (9). Finally, a total of 16 studies were involved in this systematic review and meta-analysis (**Figure 1**).

### Characteristics of included studies

A total of 16 studies conducted in African countries, including 6530 study participants, were included in this review. Among the 16 studies included, nine were from Ethiopia (40–48), four from Nigeria (19, 20, 49, 50), and one from Uganda (51), Sudan (52) and Egypt (53). All of them were followed cross-sectional study designs. Regarding assessment tools, most of them were assessed by DSM-IV (six studies) and PMSS (three studies) (**Table 1**).

**Table 1:** Characteristics of studies included in the systematic review and meta-analysis on prevalence of premenstrual syndrome and associated in Africa.

First author name (year)	Country	Participants	Study design	Tool	SZ	P %	QS	AFW [AOR(95% CI)]
Elizabeth et al, (2022) (51)	Uganda	university students	Cross-sectional	ACOG	212	28.3	8	…
Tomader (2015) (52)	Sudan	university students	Cross-sectional	DSM-IV	401	9.5	5	…
Fikru et al, (2014) (40)	Ethiopia	university students	Cross-sectional	DSM-IV	258	37	8	…
Adiss et al, (2004) (41)	Ethiopia	university students	Cross-sectional	DSM-IV	242	27	8	…
Bolurin et al, (2009) (20)	Nigeria	university students	Cross-sectional	ICD-10	404	94.8	9	
Sisay et al, (2017) (42)	Ethiopia	university students	Cross-sectional	PSST	529	36.9	9	…
Balew et al, (2023) (43)	Ethiopia	university students	Cross-sectional	PMSS	408	41.2	9	Family history of MPS: [6.34(3.47, 11.56)]; Hormonal contraceptive: [10.3(4.4, 24.1)]
Woredaw et al, (2020) (44)	Ethiopia	university students	Cross-sectional	DSM-5	386	34.7	9	Family history of PMS: [1.76(1.05, 2.92)]
Kelechi et al, (2018) (49)	Nigeria	university students	Cross-sectional	COPE	480	42.9	6	…
Natnael et al, (2022) (45)	Ethiopia	university students	Cross-sectional	PMSS	591	37.9	9	Menarche (<13 years): [2.64(1.34, 5.19)]
Abebaw et al, (2019) (46)	Ethiopia	Secoudary school students	Cross-sectional	ACOG	496	81.3	9	Menarche (<13 years): [2.68(1.32, 5.47)]
Delelegn et al, (2019) (47)	Ethiopia	university students	Cross-sectional	DSM-IV	254	66.9	9	…
A. O. et al, (2008) (19)	Nigeria	university students	Cross-sectional	DSM-IV	410	6.1	8	…
Tilahun et al, (2015) (48)	Ethiopia	Secoudary school students	Cross-sectional	DSM-IV	181	30.9	8	
Dalia et al, (2021) (53)	Egypt	university students and staffs	Cross-sectional	PMSS	668	90.5	8	…
ANTAI et al, (2004) (50)	Nigeria	Secondary school students	Cross-sectional	ICD-10	200	85.5	6	…

SZ, sample size; P, prevalence; QS, quality score; AFW, Associated factors with; AOR, adjusted odd ratio; CI, confidence interval.

### Study quality appraisals

To assess the quality of the studies we used the Joanna Briggs Institute (JBI) quality assessment criteria. All the studies involved in this review have good quality (JBI score >=5) (**Supplementary File 3**).

### Meta- analysis

**Figure 2:**
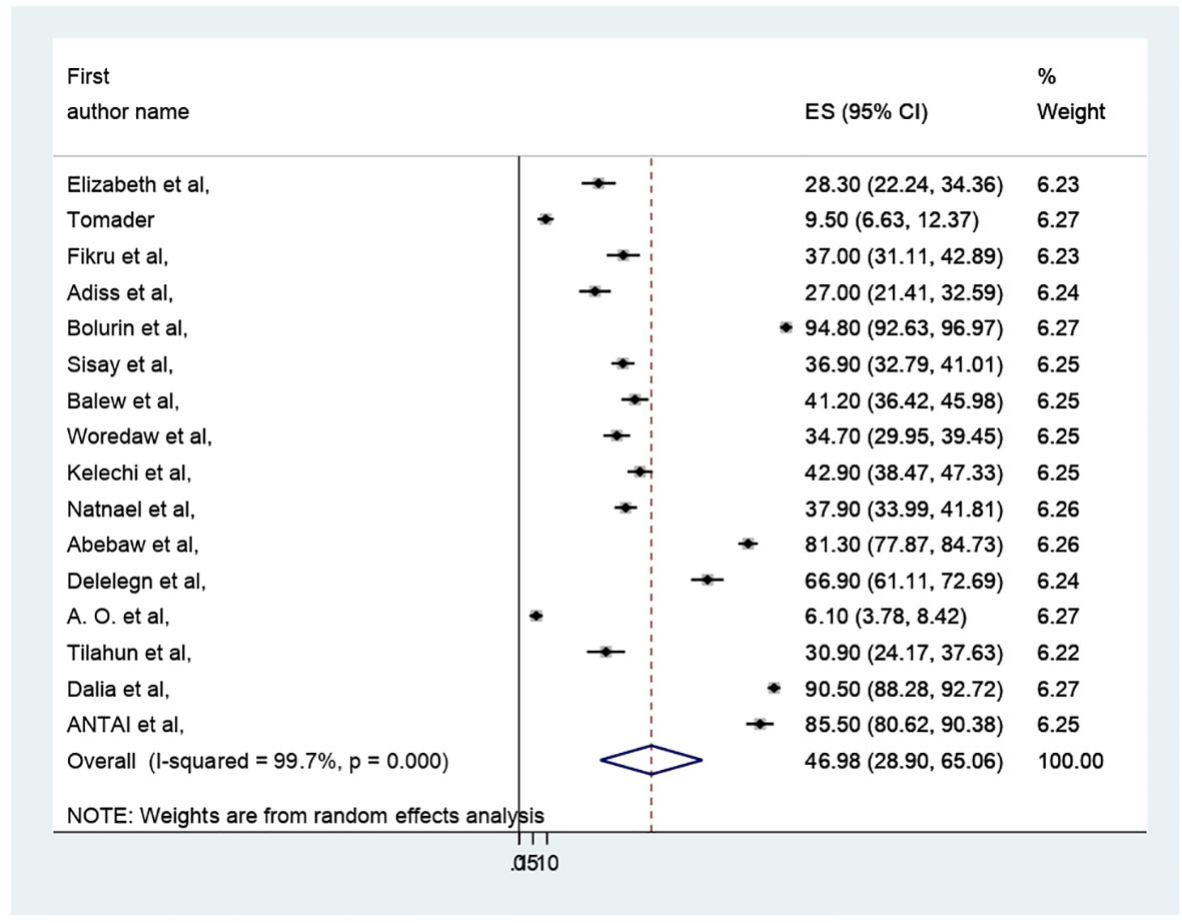
Forest plot of the pooled prevelance of PMS in Africa.

The pooled prevalence of PMS in Africa was 46.98 (95% CI: 28.9–65.06%) (**Figure 2**). Due to apparent heterogeneity across the studies, we have used a random effect model while conducting a meta-analysis (I^2 =^ 99.7%, p < 0.000).

### Publication bias

**Figure 3:**
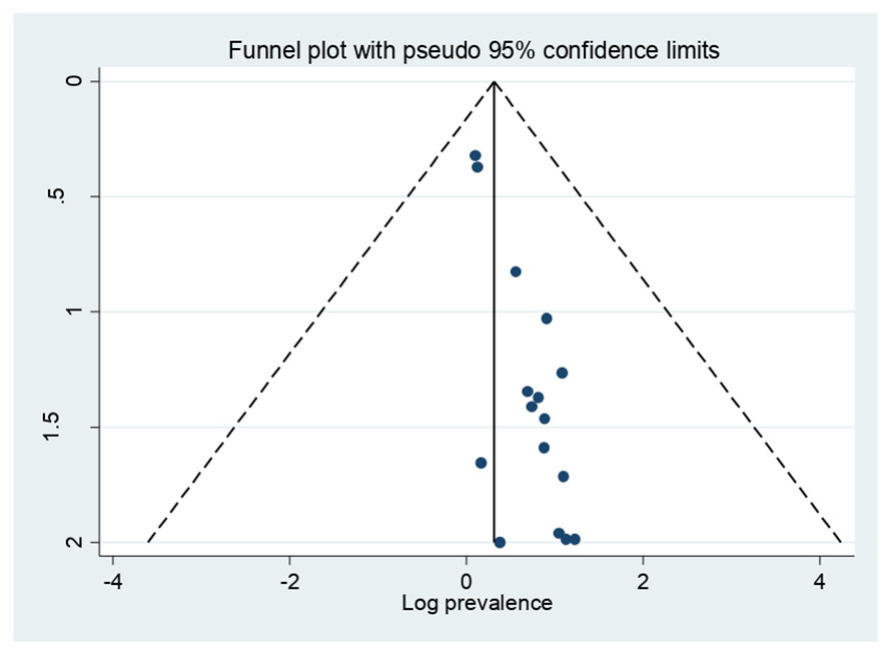
Funnel plot of PMS in Africa.

In this study, a funnel plot falls inside the triangle, which indicates the absence of publication bias (**Figure 3**), and Egger’s regression test (P = 0.346) strengthened it (**Table 2**).

**Table 2:** Egger’s test of premenstrual syndrome in Africa. A systematic review and meta-analysis.

Std_-_Eff	Coef.	Std. Err.	T	P>t	[95% Conf Interval]	
Slope	75.70633	24.63469	3.07	0.008	22.87018	128.5425
Bias	-13.18729	13.51646	-0.98	0.346	-42.17722	15.80264

### Subgroup analysis

**Figure 4:**
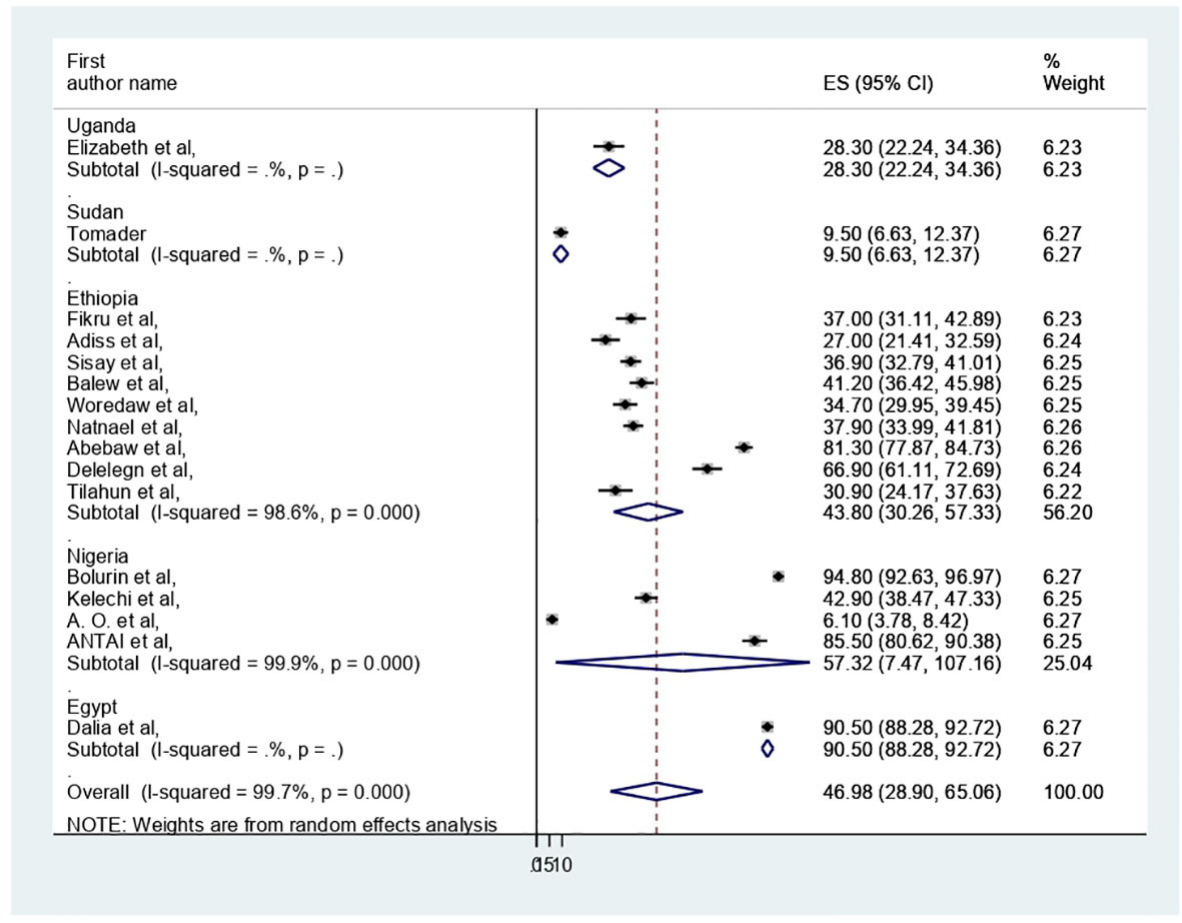
Forest plot on subgroup analysis of the pooled prevelance of PMS in Africa.

**Figure 5:**
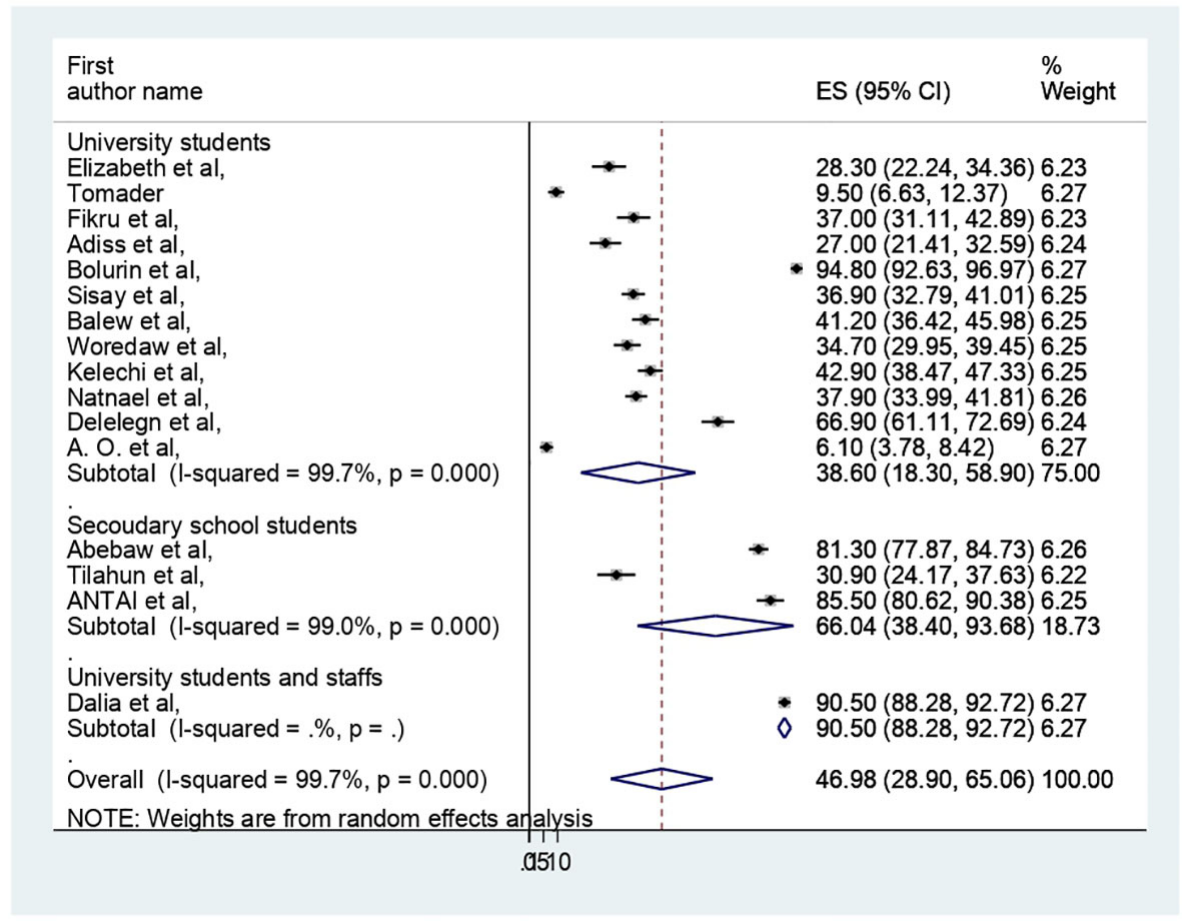
Forest plot showing different associated factors of PMS in Africa.

**Figure 6:**
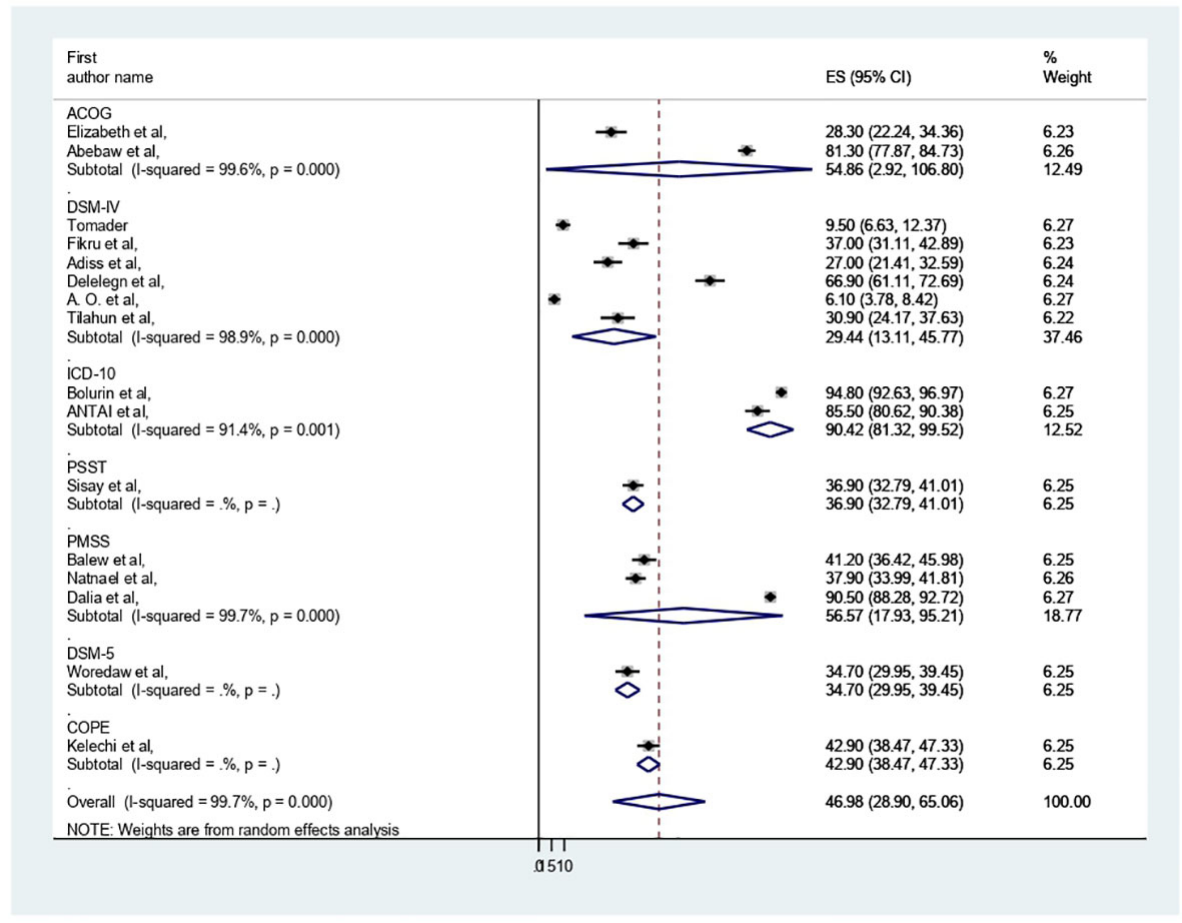
Forest plot on subgroup analysis based on the assessment tool used.

The presence of heterogeneity was confirmed (I^2 =^ 99.7%, p < 0.000). So, subgroup analysis was conducted based on study country, type of participants, and assessment tool. A higher pooled prevalence of PMS was found in Nigeria (57.32%, I^2 =^ 99.9%, p = 0.000), followed by Ethiopia (43.8%) (I^2 =^ 99.6%, p = 0.000). The study participants’ pooled prevalence of PMS among university students and secondary school students was 38.6% (I2 = 99.7%, p = 0.000) and 66.04% (I^2 =^ 99%, p = 0.000), respectively. Regarding the assessment tool, the pooled prevalence of PMS was 29.44% by DSM-IV and 56.57% by PMSS. Therefore, this result showed there was high heterogeneity among subgroups, as indicated by I^2^ (91.4 and above) and P = 0.001 (**Figures 4**–**6**), which indicate the need to conduct the sensitivity test.

### A leave-out-one sensitivity analysis

The sensitivity analysis was done to check the heterogeneity of those studies by omitting one author or one study step by step to check the effect of each study on the overall prevalence of PMS in this systematic review and meta-analysis study. As evidenced by the results, all the values are within the estimated 95% confidence-interval (CI) ranged 43.78% to 49.52%, which indicates the omission of a single study had no significant difference in the prevalence of this systematic review and meta-analysis (**Table 3**).

**Table 3:** Sensitivity analysis of PMS in Africa.

Study omitted	Estimated 95% CI	Heterogeneity	
		I^2^ (%)	P-value
Elizabeth et al,	48.22(29.41-67.03)	99.8	0.001
Tomader	49.48(31.45-67.57)	99.7	0.001
Fikru et al,	47.64(28.75-66.53)	99.8	0.001
Adiss et al,	48.31(29.48-67.13)	99.8	0.001
Bolurin et al,	43.78(26.45-61.11)	99.7	0.001
Sisay et al,	47.65(28.54-66.76)	99.8	0.001
Balew et al,	47.36(28.33-66.39)	99.8	0.001
Woredaw et al,	47.8(28.82-66.77)	99.8	0.001
Kelechi et al,	47.25(28.15-66.35)	99.8	0.001
Natnael et al,	47.58(28.42-66.75)	99.8	0.001
Abebaw et al,	44.68(25.69-63.68)	99.7	0.001
Delelegn et al,	45.65(26.74-64.56)	99.8	0.001
A. O. et al,	49.52(32.94-66.49)	99.6	0.001
Tilahun et al,	48.04(29.24-66.85)	99.8	0.001
Dalia et al,	44.07(25.97-62.16)	99.7	0.001
ANTAI et al,	44.41(25.64-63.19)	99.8	0.001

### Narrative analysis

**Figure 7:**
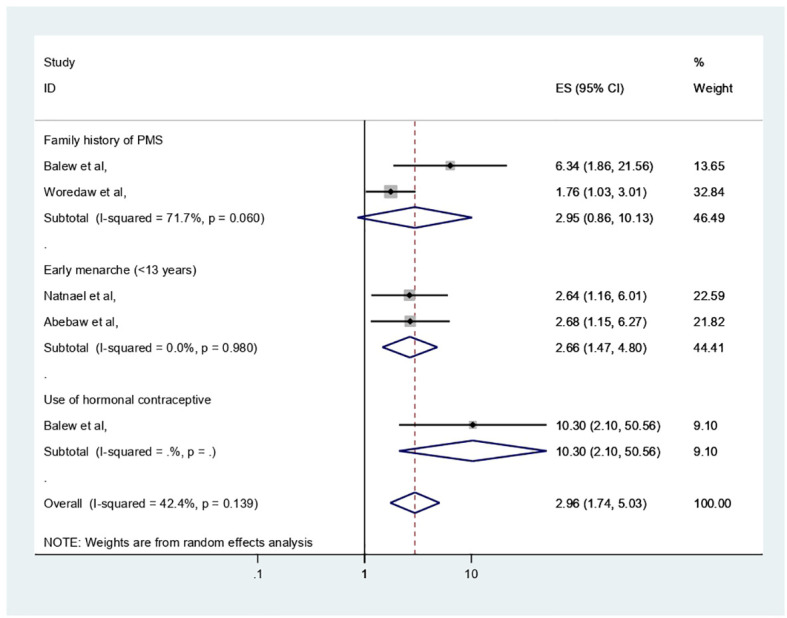
Forest plot showing different factors associated with PMS in Africa.

The extracted important factors associated with PMS among participants of reproductive age, the individual study, in Africa were provided in **Table 1** with reference to the studies analyzed in logistic regression with adjusted odd ratio. This section has narrated significant factors associated with PMS in Africa. The following results were obtained from the pooled analysis for these factors and in cases where two or more publications were present: age of menarche (<13 years) (adjusted odd ratio (AOR) = 2.66, 95% CI: 1.47–4.8) with two previous studies and within this review was significantly associated with PMS, whereas perceived family history of PMS (AOR = 2.96, 95% CI: 0.86–10.13) with two previous studies, was not significantly associated with PMS (**Figure 7**).

## Discussion

In our systematic review and meta-analysis, we synthesized 16 studies investigating the prevalence and associated factors of PMS among 6530 reproductive age participants in Africa, of whom 3068 had been screened for PMS. In this review, the pooled prevalence of PMS in Africa was 46.98 (95% CI: 28.9–65.06%). This result was in line with the previous studies of systematic reviews and meta-analyses done in Ethiopia (17, 18), India (16) and Global (7). On the other hand, it was less than the Iranian study (54). This disparity may be attributed to the study participants’ residency in Iran, but this review encompassed five nations; in Iran, 24 articles with a sample size of 9147 women of reproductive age were included. Additionally, additional assessment instruments (such as the American Psychiatric Association, Premenstrual Assessment Scale, Daily Record of Severity of Problems Chart, Researcher Made Questionnaire, Premenstrual Assessment Scale, and Hallbridge et al. questionnaire) were utilized in Iran, which were not included in this review. Further, in this analysis, DSM-IV was dominantly used for PMS assessment. This could result from differences in premenstrual syndrome screening and assessment methods’ specificity and sensitivity.

In this review, regarding subgroup analysis, the pooled prevalence of PMS among participants of reproductive age was higher in Nigeria (57.32%) compared with Ethiopia (43.8%). This finding may vary depending on the number of studies and participant sample size. For example, four research with 1494 participants were conducted in Nigeria, while nine studies with 3345 participants were conducted in Ethiopia. It could also be due to variations in research regions and assessment tools along with sociocultural differences. Additionally, we used the study participant type; among secondary school and university students, the pooled prevalence of PMS was 66.04% and 38.6%, respectively. The menarche age and participant age may be the cause, as shown by the results of the earlier Iranian review (54).

In terms of factors associated with PMS, they were extracted in the individual study in Africa with reference to the studies analyzed in logistic regression with an adjusted odd ratio. However, most studies were analyzed by ANOVA, and some of them involved linear logistic regression.

The important variables linked to PMS have been discussed in this section, and in cases where two or more publications were present, our review identified that age at menarche and family history of PMS were positively and negatively associated with PMS. According to this finding, early menarche (<13 years) (AOR = 2.66, 95% CI: 1.47–4.8) was a risk factor for PMS. The results of this study could be explained by the earlier menarche age being linked to the early development of ovarian functions and ovulation with steroid hormone fluctuations at such a young age with less physical and psychological maturity, which may cause PMS manifestations; however, the earlier Ethiopian meta-analysis did not support this theory (17). In contrast, perceived family history of PMS (AOR = 2.96, 95% CI: 0.86–10.13) was not significantly associated with PMS in this analysis. The severe level of premenstrual dysphoric disorder’s heritability in the United States is unknown. However, Premenstrual symptoms have heritability estimates ranging from 30% to 80% (6).

Regarding this study, all participants were secondary school and university students. This is a productive age group, so the ministry of education should incorporate the module into the curriculum and provide training about premenstrual symptoms and their management. We recommend more representative samples, or rather, a cross-sectional study design, be used in future research that concentrates on a more precise diagnosis.

## Limitation

The limitation of this review is that it included only studies published in English that were cross-sectional studies since there were no studies conducted with other study designs and a small number of articles were included.

## Conclusion

In this review, the pooled prevalence of PMS in Africa was high. Among factors, the early age of menarche was a risk factor for PMS. This finding might help the stakeholders (mental health policy makers, administrators, and mental health professionals) to address prevention, early screening, and management of PMS among reproductive-age women and to give attention to more vulnerable bodies.

## Data availability statement

The original contributions presented in the study are included in the article/**Supplementary Material**. Further inquiries can be directed to the corresponding author.

## Author contributions

FA: Conceptualization, Data curation, Methodology, Software, Validation, Writing – original draft, Writing – review & editing. MM: Data curation, Writing – review & editing. GiM: Data curation, Writing – review & editing. GN: Writing – review & editing. TT: Writing – review & editing. SF: Writing – review & editing. GR: Writing – review & editing. JS: Writing – review & editing. GeM: Writing – review & editing. DA: Writing – review & editing. TN: Writing – review & editing.
