# Supplementary material for: Prevalence of premenstrual syndrome and its associated factors in Africa: a systematic review and meta-analysis
Source: Front Psychiatry. 2024 Jan 31;15:1338304. doi: 10.3389/fpsyt.2024.1338304 (PMC10865226; doi:10.3389/fpsyt.2024.1338304)
Supplement: Supplementary file 1 [file DataSheet_1.docx]

**Searching strategy for data base**

EMBASE

((('prevalence'/exp OR 'prevalence' OR 'epidemiology'/exp OR 'epidemiology' OR 'magnitude'/exp OR 'magnitude') AND ('premenstrual syndrome'/exp OR 'premenstrual syndrome') OR 'premenstrual dysphoric disorder'/exp OR 'premenstrual dysphoric disorder') AND 'associated factors' OR 'risk factors'/exp OR 'risk factors') AND ('women'/exp OR 'women') AND ('africa'/exp OR 'africa') AND [embase]/lim

**Medline**

((('prevalence'/exp OR 'prevalence' OR 'epidemiology'/exp OR 'epidemiology' OR 'magnitude'/exp OR 'magnitude') AND ('premenstrual syndrome'/exp OR 'premenstrual syndrome') OR 'premenstrual dysphoric disorder'/exp OR 'premenstrual dysphoric disorder') AND 'associated factors' OR 'risk factors'/exp OR 'risk factors') AND ('women'/exp OR 'women') AND ('africa'/exp OR 'africa') AND [embase]/lim AND [medline]/lim

**Cochrane library**

(“Prevalence” OR “epidemiology” OR “magnitude” AND “premenstrual syndrome” OR “premenstrual dysphoric disorder” AND “associated factors” OR “risk factors” AND “women” AND “Africa”) in Title Abstract Keyword

**Scopus**

TITLE-ABS-KEY "Prevalence" OR "epidemiology" AND "premenstrual syndrome" OR "premenstrual dysphoric disorder" AND "associated factors" OR "risk factors" AND "Africa"

**Table S 3 (Supplementary File 3):** Quality assessment of premenstrual syndrome and its associated factors in Africa included studies in this systematic review and meta-analysis.

| First author name (year) | Q1 | Q2 | Q3 | Q4 | Q5 | Q6 | Q7 | Q8 | Q9 | Total score (9%) |
| --- | --- | --- | --- | --- | --- | --- | --- | --- | --- | --- |
| Elizabeth et al, (2022) | Y | Y | Y | Y | Y | Y | Y | Y | N | 8 |
| Tomader | NR | YNR | NR | Y | Y | Y | Y | Y | NR | 5 |
| Fikru et al, (2014) | Y | Y | N | Y | Y | Y | Y | Y | Y | 8 |
| Adiss et al, (2004) | Y | Y | N | Y | Y | Y | Y | Y | Y | 8 |
| Bolurin et al, (2009) | Y | Y | Y | Y | Y | Y | Y | Y | Y | 9 |
| Sisay et al, (2017) | Y | Y | Y | Y | Y | Y | Y | Y | Y | 9 |
| Balew et al, (2023) | Y | Y | Y | Y | Y | Y | Y | Y | Y | 9 |
| Woredaw et al, (2020) | Y | Y | Y | Y | Y | Y | Y | Y | Y | 9 |
| A. O. et al, (2008) | Y | Y | NR | Y | Y | Y | Y | Y | Y | 8 |
| Natnael et al, (2022) | Y | Y | Y | Y | Y | Y | Y | Y | Y | 9 |
| Abebaw et al, (2019) | Y | Y | Y | Y | Y | Y | Y | Y | Y | 9 |
| Delelegn et al, (2019) | Y | Y | Y | Y | Y | Y | Y | Y | Y | 9 |
| Tilahun et al, (2015) | Y | Y | N | Y | Y | Y | Y | Y | Y | 8 |
| Dalia et al, (2021) | Y | Y | N | Y | Y | Y | Y | Y | NR | 8 |
| ANTAI et al, (2004) | Y | Y | NR | Y | Y | NR | Y | Y | NR | 6 |
| Kelechi et al, (2018) | NR | NR | NR | Y | Y | Y | Y | Y | Y | 6 |

**Key:** **Y**= Yes; **N**= No; **NR**= Not reported, **NA**=Not appropriate

**Question codes:**

1. Was the sample frame appropriate to address the target population?

2. Were study participants sampled in an appropriate way?

3. Was the sample size adequate?

4. Were the study subjects and the setting described in detail?

5. Was the data analysis conducted with sufficient coverage of the identified sample?

6. Were valid methods used for the identification of the condition?

7. Was the condition measured in a standard, reliable way for all participants?

8. Was there appropriate statistical analysis?

9. was the response rate adequate, and if not, was the low response rate managed appropriately?
